# An Intelligent Identification Method for the Processing Degree of Stir-Fried *Cannabis fructus* Based on Multi-Source Information Fusion and Machine Learning

**DOI:** 10.3390/foods15162817

**Published:** 2026-08-12

**Authors:** Kaiwen Chen, Hao Zhang, Ge Tian, Jinjuan Wu, Tulin Lu, De Ji, Chuanshan Jin, Deling Wu, Xiaoli Wang

**Affiliations:** 1 Department of Pharmacy, Anhui University of Chinese Medicine, Hefei 230012, China; ckw200136@163.com (K.C.); 19855535017@163.com (H.Z.); 13513227572@163.com (G.T.); 18110493165@163.com (J.W.); jinchuanshan@ahtcm.edu.cn (C.J.); 2 Department of Pharmacy, Nanjing University of Traditional Chinese Medicine, Nanjing 230012, China; lutuling2005@126.com (T.L.); de.ji@njucm.edu.cn (D.J.); 3 Heritage Base of TCM Processing Technology of NATCM, Anhui University of Chinese Medicine, Hefei 230012, China; 4 Anhui Province Key Laboratory of Traditional Chinese Medicine Decoction Pieces of New Manufacturing Technology, Hefei 230012, China; 5 Bozhou Key Laboratory of Biosynthesis of Effective Components of Medicinal Plants, Bozhou University, Bozhou 236800, China

**Keywords:** *Cannabis fructus*, machine vision, machine learning, electronic nose, multi-source information fusion

## Abstract

*Cannabis fructus* (CF) has been used as a functional food raw material. The rapid identification of its processing degree is vital for ensuring consistency of product quality, achieving standardized processing, and maintaining market authenticity. This study established an intelligent identification method for the processing degree of stir-fried CF based on multi-source information fusion and machine learning. Color (L, a, b) and odor signals of the samples were collected through machine vision and electronic nose systems. The total contents of fatty oil and trigonelline were determined. Among the evaluated models, XGBoost demonstrated superior performance, achieving near-perfect training accuracy (93.62%) and test accuracy (91.67%). Shapley Additive Explanations analysis revealed that color and signals from specific odor sensors were key distinguishing features. The color exhibited a gradual transition to light yellow, and total fat and oil content showed a likely positive correlation with a and b values. The content of senna alkaloids tended to reduce as frying proceeded to greater intensity. This work preliminarily uncovered the correlations among color, gas characteristics, and quality, and could provide new insights for intelligent monitoring of the preparation procedure.

## 1. Introduction

*Cannabis fructus* (CF) is the mature, dried fruit of *Cannabis sativa* L. of the Cannabaceae family [1,2,3], and is a common food ingredient in traditional Chinese dietary and healthcare practice [4]. As a nutrient-rich ingredient, CF has long been applied in improving intestinal comfort. Recent studies indicate that CF can help regulate blood lipids, reduce inflammation, and exert antioxidant activity, which may also improve cognitive function. This shows that it has good potential as a functional food ingredient [5,6,7,8]. Fried CF is a common form of edible hemp seed obtained by light frying until it turns light-yellow. It not only helps cleanse the intestines but also supplies nutrients. Heating processes can also mitigate the bitterness of CF and improve flavor. Currently, the processing endpoint is still mainly determined by manual and experience-based sensory methods, combined with physical and chemical tests. This method has limitations, such as unclear standards, high subjectivity, and substantial variability due to personal experience, resulting in uneven product quality and significant variation in the appearance and flavor of the commercially available products. Thus, a practical problem arises: how to establish a standardized, objective, and rapid method for identifying the processing degree of fried CF?

In food processing and quality control, traditional methods reliant on manual experience face challenges such as low efficiency and inconsistency. With the rapid advancement of electronic sensing technology and deep learning algorithms, intelligent detection methods have been gradually introduced into food quality evaluation systems [9,10]. Machine learning can systematically extract color and odor characteristics of products at different cooking stages [11], achieve precise classification, and continuously optimize recognition accuracy through continuous training, effectively addressing the challenges of quantitatively assessing the degree of cooking. For instance, technologies like electronic eyes and machine learning can quickly identify different types of freshly processed coffee beans.

The quality assessment of traditional Chinese medicine decoction pieces, such as fried CF, currently relies heavily on the experiential judgment of seasoned practitioners to determine their processing degree. While this traditional approach is effective, it suffers from limitations, including high subjectivity, difficulty in quantification, and poor reproducibility [12,13]. To overcome these challenges, multi-information fusion strategies have been introduced in recent studies. By combining machine vision and electronic nose data, these approaches offer a more comprehensive view of the manufacturing process [14], overcoming the limitations of single-source analysis. Grounded in the traditional Chinese medicine processing concept that color, odor, and taste serve as critical criteria for assessing the processing degree, this study establishes a multi-dimensional fusion-based intelligent identification model that integrates color, odor, and intrinsic components.

In the field of intelligent food recognition, machine learning methods based on multi-source information fusion are becoming key in solving complex classification problems [12,15]. Various machine learning algorithms are now widely used to classify samples with distinct features such as color and smell, characteristics also present in fried CF. Compared to deep learning models that require huge amounts of data, the sample size of 180 in this study is insufficient to support the effective training of models such as CNNs, so they were not included in the comparison. However, despite growing interest in multi-source fusion for food and TCM quality evaluation, several notable gaps remain in the current literature. First, most existing studies prioritize classification accuracy as the primary outcome, with relatively few efforts devoted to linking external sensory features to pharmacopeia-relevant internal quality markers; the quantitative relationship between appearance/odor and intrinsic constituents thus remains insufficiently characterized for most processed herbs. Second, many fusion models function as black-box systems. The lack of interpretability inherently limits their practical adoption in food quality control, where decision-making transparency is essential, and such feature-level attribution analyses remain underemphasized in TCM processing research, further hindering acceptance in industrial settings where practitioners rely on clear decision rationale. Third, systematic head-to-head comparisons of multiple machine learning algorithms under standardized cross-validation frameworks are relatively limited for seed-class processed herbs, making generalizable recommendations for model selection difficult. To address these gaps, the present study comprehensively evaluated six machine learning algorithms and employed SHAP (Shapley Additive Explanations) to quantitatively analyze the contribution mechanisms of key features to classification decisions [16], thereby establishing a robust, efficient, and transparent intelligent identification system for the processing degree of fried CF.

To address these gaps, this study introduces a multi-source information fusion strategy that combines machine vision (Lab color space) and an electronic nose (odor sensor array). Unlike single-source approaches, this fusion captures complementary sensory dimensions that collectively reflect the physicochemical changes occurring during frying (Maillard reaction, caramelization, volatile compound generation). Furthermore, we integrate interpretable machine learning—specifically the XGBoost algorithm coupled with SHAP (Shapley Additive Explanations) analysis—to not only classify the four processing degrees but also quantify the contribution of each color and odor feature to the classification decision. Among the various chemical constituents of CF, we deliberately focused on two representative indicators: total fatty oil and trigonelline.

Among various components of *Cannabis fructus*, total fatty oil and trigonelline were selected as quality indicators consistent with national standards for stir-fried formula granules [17]. Total fatty oil accounts for over 40% of raw CF. Seed rupture during stir-frying facilitates oil release, and its content variation directly reflects thermal processing degree and nutrient retention. Trigonelline is a widespread alkaloid in seed medicinal herbs. Existing research confirms it acts as an NAD^+^ precursor that benefits skeletal muscle health; its serum concentration declines in patients with sarcopenia, indicating valuable physiological activity [18]. Under our fixed stir-frying conditions, the detectable extractable trigonelline content of hemp seed generally increases with moderate heating but declines after sustained high-temperature treatment. Such variation may help distinguish over-stir-fried samples. CF also contains polyphenols and lignanamides, but their quantification requires costly LC-MS analysis and cannot meet the demand of rapid factory preliminary screening. Gravimetry and HPLC for the two selected markers are mature, low-cost, routine detection methods. Comprehensive multi-component profiling will be supplemented in follow-up research.

Collectively, this work makes the following contributions to the existing literature. First, it provides a complete multi-source fusion workflow tailored to stir-fried *Cannabis fructus* processing degree identification, offering empirical data and methodological reference for this specific herb. Second, it establishes preliminary quantitative correlations between non-destructive sensory features and internal quality markers, which may inform the design of rapid on-site screening protocols. Third, the combination of systematic algorithm benchmarking and SHAP-based interpretability analysis yields both performance reference and feature-level insights, which we hope will serve as a useful methodological reference for similar studies on processed TCM materials.

## 2. Methods

### 2.1. Experimental Materials and Reagents

Fifteen batches of raw CF were supplied by Anhui Huaxiu Pharmaceutical Co., Ltd., (Bozhou, China) all identified by Associate Professor Yang Qingshan from Anhui University of Chinese Medicine as dried mature fruits of *Cannabis sativa* L. (Cannabaceae). These 15 independent raw material batches were harvested from three major producing regions of CF in China, including Shanxi, Henan, and Heilongjiang, covering the majority of the country’s primary cultivation areas. Complete batch numbers and origin information are documented in Supplementary Table S1 for reference. For experimental analysis, 15 raw material batches were each subjected to three independent stir-frying replicates covering four distinct processing degrees, which generated a total of 180 test samples. Petroleum ether (Shanghai, China LOT: K2420638), methanol (Shanghai, China LOT: 25111101G7Z2), sodium dodecyl sulfate (Shanghai, China LOT: C15315007), and acetic acid Shanghai, China (LOT: 2024091801). Petroleum ether was of analytical purity; water was Wahaha Pure Water (Hangzhou Wahaha Group Co., Ltd., Hangzhou, China). Trigonelline (Chendu, China LOT: DST240410-040, purity ≥ 98%).

### 2.2. Preparation of CF Samples with Different Frying Degrees

According to the processing procedure outlined in the 2025 edition of the Chinese Pharmacopeia for CF, crude medicinal materials were collected, followed by removal of impurities and the outer pericarp to obtain raw CF slices. Stir-frying was performed using a 1000 W Midea induction cooker (IDEA GmbH, Pfalzgrafenweiler-Bösingen, Germany). The wok was preheated for 1 min before sample loading. Real-time temperature data were collected by thermocouples at 30 s intervals. The temperature was maintained at 180 ± 5 °C during the whole heating process under constant stirring to achieve homogeneous heating of samples. For each treatment batch, 200.0 g of raw CF slices were stir-fried for 8 min, 15 min, and 22 min, respectively, yielding three groups of samples with distinct thermal processing degrees: insufficiently stir-fried, moderately stir-fried, and excessively stir-fried.

### 2.3. Obtaining Color Information Based on the Machine Vision Lab Model

Image capture was performed using a Sony A7C2 full-frame digital camera and a Sony FE 50 1.4 GM lens (Sony Corporation, Tokyo, Japan). Manual focus mode was used, white balance was set to 6600 K and further calibrated using a standard color card, with shutter speed at 1/200, aperture at F10, and ISO set to 100. Images were captured in RGB color mode at a resolution of 4672 × 4672 and saved in JPG format. Images were captured inside a closed light chamber with internal dimensions of 40 cm × 40 cm × 40 cm. A standard D65 ring LED light source with an illumination intensity of 800 lx was installed within the sealed chamber to eliminate ambient stray light interference. Python 3.10 was used to extract the Lab color space of the main body of the fried CF image. A total of 180 sample groups derived from 15 batches of fried CF pieces with varying processing degrees were collected. For each of the four types—raw, insufficiently fried, moderately fried, and excessively fried—15 g of pieces were placed in an imaging chamber for image acquisition. Each sample was photographed in three parallel sets, resulting in a total of 540 images. Results were calculated as the average of these parallel determinations [19,20].

#### Lab Color Space Extraction

Acquisition and digital processing of color information were performed using a machine vision method. All samples were imaged using a high-resolution digital camera inside a stable light chamber illuminated with a standard D65 light source and a 45°/0° observation angle. Image processing was performed using Python 3.10 and the OpenCV library: raw captured images were read and uniformly converted to the three-channel BGR color space, a color image threshold segmentation method with defined BGR threshold ranges was adopted to generate binary masks, and the identified white background pixels were filled with black to achieve background suppression. The region of interest (ROI) of *Cannabis fructus* samples was delineated by all non-zero color pixels in the processed image to exclude invalid background pixels; no image enhancement operations such as filtering or contrast stretching were applied during preprocessing to fully preserve the original pixel color information, and within the ROI, the cv2.cvtColor() function in OpenCV was invoked to complete BGR-to-Lab color space conversion, based on which Lab color feature parameters were finally calculated [19,21].

### 2.4. Acquisition of Odor Information Based on Electronic Nose Technology

The electronic nose (PEN3, Airsense Analytics, Schwerin, Germany) contains ten metal oxide semiconductor sensors with specific sensitivity to different volatile compounds. Sensor W1C is sensitive to aromatic compounds; W5S responds to nitrogen oxides; W3C is sensitive to ammonia and aromatic compounds; W6S is mainly sensitive to hydrides; W5C targets short-chain alkanes and aromatic compounds; W1S is sensitive to methyl compounds; W1W responds to sulfur-containing organics; W2S is sensitive to alcohols, aldehydes and ketones; W2W detects sulfur chlorides and aromatic compounds; and W3S is sensitive to long-chain alkanes. A total of 180 powder samples of CF with four processing degrees were separately placed in 50 mL beakers. After sealing, the samples were allowed to equilibrate for 24 h and were then analyzed using a PEN 3 electronic nose system (Airsense Analytics, Schwerin, Germany) [22]. The sampling needle was inserted into the beaker to collect odor data. Clean air was used as a blank reference for baseline correction, and a sensor self-cleaning time of 150 s was set to eliminate baseline drift. Sample preparation time was set at 5 s, while the analysis time was set at 150 s. Data analysis was performed during the stable signal phase of the sensors, with three parallel samples per group. Data processing was conducted using WinMuster v1.6.2 [23].

### 2.5. Establishment of a Classification Model

The final machine learning dataset was constructed with a clear hierarchical experimental design. Based on 15 independent batches of CF originating from three producing regions, each batch was subjected to three parallel stir-frying replications, and every replication covered four distinct processing degrees (Raw product, Insufficient frying degree product, Moderate frying degree product, Excessive degree of frying product). This design yielded a total of 180 independent samples. Three independent parallel tests were performed for each stir-frying replication: each parallel test included one machine vision acquisition (with three repeated images for average Lab value calculation) and one electronic nose detection, corresponding to one independent sample in the dataset containing paired three-dimensional Lab color features and 10-dimensional electronic nose sensor features. Overall, the full dataset included 180 samples (45 samples per processing degree), which were used for subsequent model training, hyperparameter tuning, and validation.

The 13 features were chosen to capture stir-frying-induced changes: three Lab channels quantify color gradients from Maillard and lipid browning, while 10 e-nose sensors cover volatile fingerprints. Their integration follows traditional “color–odor” quality assessment, jointly reflecting surface heating and internal chemical transformations. These low-dimensional, non-redundant features preserve subtle quality variations for robust classification and on-site quality control.

In this study, a classification model was constructed based on standardized data obtained from machine vision color feature extraction and electronic nose odor information acquisition. Each sample was represented by a 13-dimensional feature vector, comprising three-dimensional Lab color features and 10-dimensional electronic nose sensor response features. The dataset included a total of 180 samples encompassing the four processing degrees—raw products, under-processed, appropriately processed, and over-processed products—with 45 samples per category.

Six machine learning algorithms with distinct internal mechanisms were selected for comparative classification evaluation according to uniform stratified sampling and cross-validation rules, including Logistic Regression (linear discriminant baseline), KNN (instance-based learning), Naive Bayes (lightweight probabilistic classifier), Random Forest (bagging ensemble), XGBoost, and CatBoost (two optimized gradient-boosting ensemble algorithms). The screening standards of classifiers include full coverage of different algorithm paradigms, stable anti-overfitting performance on small-sample datasets, native compatibility with SHAP interpretable analysis, low computing cost for industrial deployment, and wide application in herbal medicine multi-sensor fusion detection. Other mainstream ensemble algorithms (LightGBM, AdaBoost, conventional GBDT) and deep learning architectures (CNN, LSTM) were excluded from the comparison: gradient-boosting algorithms represented by XGBoost and CatBoost already cover core tree ensemble frameworks, and adding similar gradient-boosting models would lead to redundant results; AdaBoost exhibited severe overfitting against the noise of electronic nose signals in preliminary tests. In addition, deep learning models require large-scale samples and high computational consumption, which are inconsistent with the small-sample characteristic and lightweight on-site monitoring target of this study. A stratified hold-out split combined with internal stratified 10-fold cross-validation was adopted for this four-class classification task. The dataset was partitioned into training and independent test subsets via a single stratified 8:2 hold-out split, which reserves a completely unseen test partition for unbiased final classification evaluation and mitigates random partitioning bias. This global split was not used as the sole validation strategy: the held-out test set was entirely excluded from model training and hyperparameter tuning to prevent data leakage and guarantee unbiased generalization assessment, while stratified 10-fold cross-validation was performed exclusively within the 80% training subset throughout hyperparameter optimization to fully exploit limited samples, reduce overfitting risks, and obtain reliable tuning outcomes [24]. Hyperparameter tuning was implemented via GridSearchCV for exhaustive parameter traversal across predefined candidate ranges. A variety of regularization hyperparameters (e.g., l2_leaf_reg, random_strength) were included within the search space to alleviate overfitting issues on the small-sample dataset. Stratified 10-fold cross-validation was conducted within the training set for all search rounds. The hyperparameter combination yielding the highest average cross-validation classification accuracy was identified as the optimal configuration for each classifier. Model performance was evaluated using six metrics: accuracy, specificity, F1-score, recall, precision, and the area under the curve (AUC). Concise metric definitions tailored to this four-class task are supplemented as follows. Accuracy refers to the overall proportion of correctly classified samples. Macro precision and macro recall represent unweighted average precision and true positive detection rate across all four stir-frying groups, respectively. Macro F1-score, the harmonic meaning of macro precision and macro recall, balances class-wise performance without sample-size weighting. Macro AUC averages one-versus-rest sub-classification AUC values as a threshold-independent discriminative index; AUC values range from 0 to 1, where values closer to 1 signify superior model discrimination capacity [25]. Additionally, confusion matrix visualization was used to assess the classification performance of the model. Based on methods used in previous studies, SHAP models for interpretable machine learning were used to facilitate global interpretation. Shapley values for each feature in the model were calculated to rank feature importance, enhancing model trustworthiness and transparency. All machine learning models were implemented on the Jupyter Notebook platform using Python (v3.10) and the scikit-learn library. SHAP values for the samples were derived using the SHAP library. Deep learning models (e.g., CNN and LSTM) were not compared in this study due to data and application constraints. Specifically, deep learning methods require large-scale samples to avoid overfitting and ensure favorable generalization capability, which is incompatible with the small-sample and low-dimensional feature characteristics of the present dataset. Moreover, this study aimed to establish an efficient and portable model for on-site quality control of processing products. Classical machine learning algorithms with concise structures and high computational efficiency can achieve promising classification performance while better meeting practical industrial deployment requirements, thereby avoiding the unnecessary complexity of deep learning frameworks. Hyperparameter optimization for all six classifiers was performed via stratified 10-fold cross-validation. The optimal hyperparameter combination screened for each model is summarized in Table 1.

### 2.6. Quantitative Analysis of Total Fatty Oil and Trigonelline

#### 2.6.1. Determination of Total Fatty Oil

A total of 1.0 g powder of each processed *Cannabis fructus* sample was accurately weighed, an appropriate volume of petroleum ether (60–90 °C) was added, and reflux extraction was performed for 5 h using a Soxhlet extractor (Infitek, Shanghai, China). The extract was transferred to a pre-dried and constant-weight evaporating dish, evaporated to dryness in a low-temperature water bath, dried at 100 °C for 60 min, and then cooled in a desiccator and weighed to calculate the total fatty oil content. Three parallel replicate extractions were carried out for every single sample to reduce experimental error.

#### 2.6.2. HPLC Determination of Trigonelline

##### Chromatographic Conditions

Chromatographic conditions: Octadecylsilane (ODS)-bonded silica was used as the stationary phase (column length: 250 mm, internal diameter: 4.6 mm, particle size: 5 μm); the mobile phase comprised a mixture of methanol, a 0.05% sodium dodecyl sulfate solution, and glacial acetic acid, prepared in a ratio of 20:80:0.1. The flow rate was set to 0.5 mL/min; injection volume was 20 μL; column temperature was maintained at 30 °C; and detection wavelength was established at 265 nm [26,27].

##### Preparation of Reference and Test Solutions

Reference solution: Accurately weigh trigonelline standard, dissolve with 50% methanol to prepare a series of calibration standard solutions with concentrations of 3.00, 6.00, 7.50, 15.00, 30.00, 60.00 μg/mL. Test solution: Precisely weigh 0.4 g sample powder, add 25 mL 50% methanol, ultrasonically extract (250 W, 40 kHz) for 30 min, and filter to obtain the test solution. Each sample was prepared and measured in three parallel replicates.

##### Calibration Curve and Method Validation

Calibration information: The peak area (Y) was taken as the ordinate and the standard concentration (X) as the abscissa for linear regression. The regression equation was Y = 0.3907X + 0.1245, with a correlation coefficient R^2^ = 0.9998, showing satisfactory linearity over the range of 3.00–60.00 μg/mL. A precision test was conducted with six consecutive injections of the reference solution, giving an RSD value of 1.67%. Stability test: The test solution was analyzed at different time points within 24 h, and the RSD of trigonelline peak areas was 2.37%. Repeatability was evaluated using six independently prepared test solutions from one batch of samples, with an RSD of 0.96%. For spike–recovery assays, the average recovery of trigonelline reached 99.51% and the corresponding RSD was 2.46%.

### 2.7. Data Analysis

Statistical analysis and data visualization were performed using Origin 2024 and SIMCA14.1 software. One-way analysis of variance (one-way ANOVA) was first carried out to identify overall differences in total fatty oil and trigonelline contents among the four groups (Raw product, Samples with an insufficient frying degree, Samples with a moderate frying degree, Samples with an excessive degree of frying). When ANOVA showed significant intergroup differences, an independent two-sample *t*-test was used as the post hoc method for pairwise comparisons. The significance level for all statistical analyses was set at α = 0.05, where *p* < 0.05 represented a significant difference and *p* < 0.01 indicated an extremely significant difference. Spearman’s correlation analysis was conducted to explore correlations between Lab color parameters, electronic nose signals, and the contents of intrinsic active ingredients.

## 3. Results and Discussion

### 3.1. Analysis of the Machine Vision Lab Color Space Model

**Figure 1:**
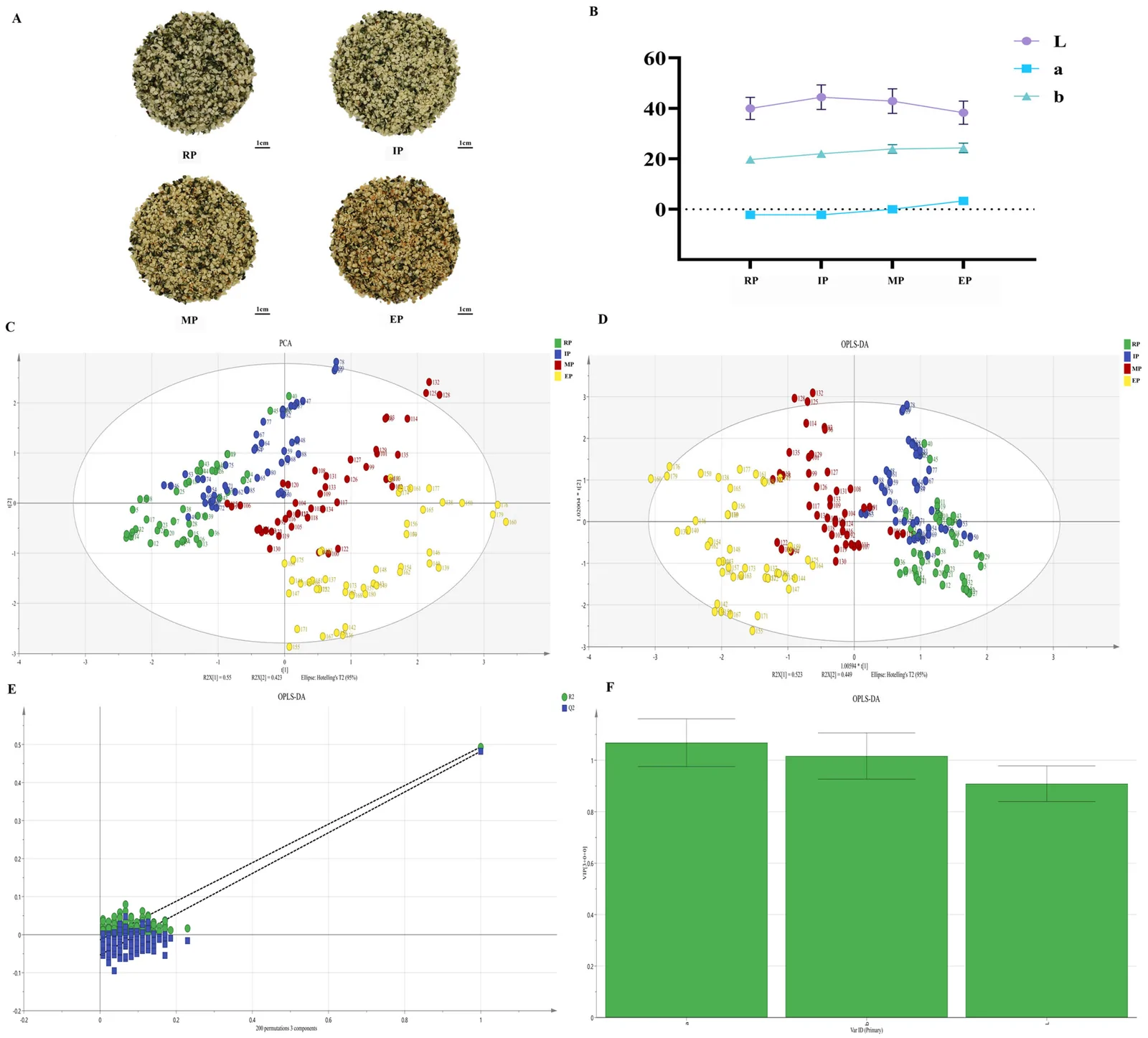
Machine vision experiment result chart. (**A**) Samples of CF at different processing degrees. (**B**) Lab color changes in CF caused by different processing degrees. (**C**): Extraction of PCA diagram by Lab color. (**D**,**E**) Extraction of OPLS-DA analysis diagram by Lab color. (**F**): Lab color extraction VIP value map.

CF with different processing degrees exhibited distinct differences in appearance and color (Figure 1A). Machine vision technology was used to extract Lab color features from samples across the four processing categories (RP, IP, MP, and EP). Traditional multivariate statistical analysis was conducted using principal component analysis (PCA) and orthogonal partial least squares-discriminant analysis (OPLS-DA). The results showed that the Lab color space achieved a cumulative variance contribution rate of 95%, indicating its effectiveness in retaining the core information of color features. However, in PCA, samples from the four processing degrees showed poor clustering performance. Specifically, the color features of RP and IP displayed a high degree of overlap, making clear differentiation difficult (Figure 1C). OPLS-DA results further confirmed that although L and b values exhibited some differences among the four sample types, the overall separation performance still failed to meet the requirement for accurate discrimination (Figure 1D–F).

Analysis of dynamic changes in Lab color parameters revealed that the influence of processing degree on CF was mainly concentrated in regular fluctuations of the L and b values (Figure 1B). As processing time increased, the b value initially decreased before subsequently rising. The L value initially rose, reaching a peak at 8 min of stir-frying, and then gradually decreased with prolonged processing time. This change pattern correlates closely with physicochemical alterations occurring during processing; in the initial heating stage, the evaporation of internal moisture and the melting and exudation of oils from CF increased sample brightness. With the prolongation of processing time, heat accumulation triggered non-enzymatic browning processes, such as the Maillard reaction and caramelization, generating substantial brownish substances [28,29] and leading to a decrease in the L value and a deepening of color.

### 3.2. Electronic Nose Odor Feature Analysis

**Figure 2:**
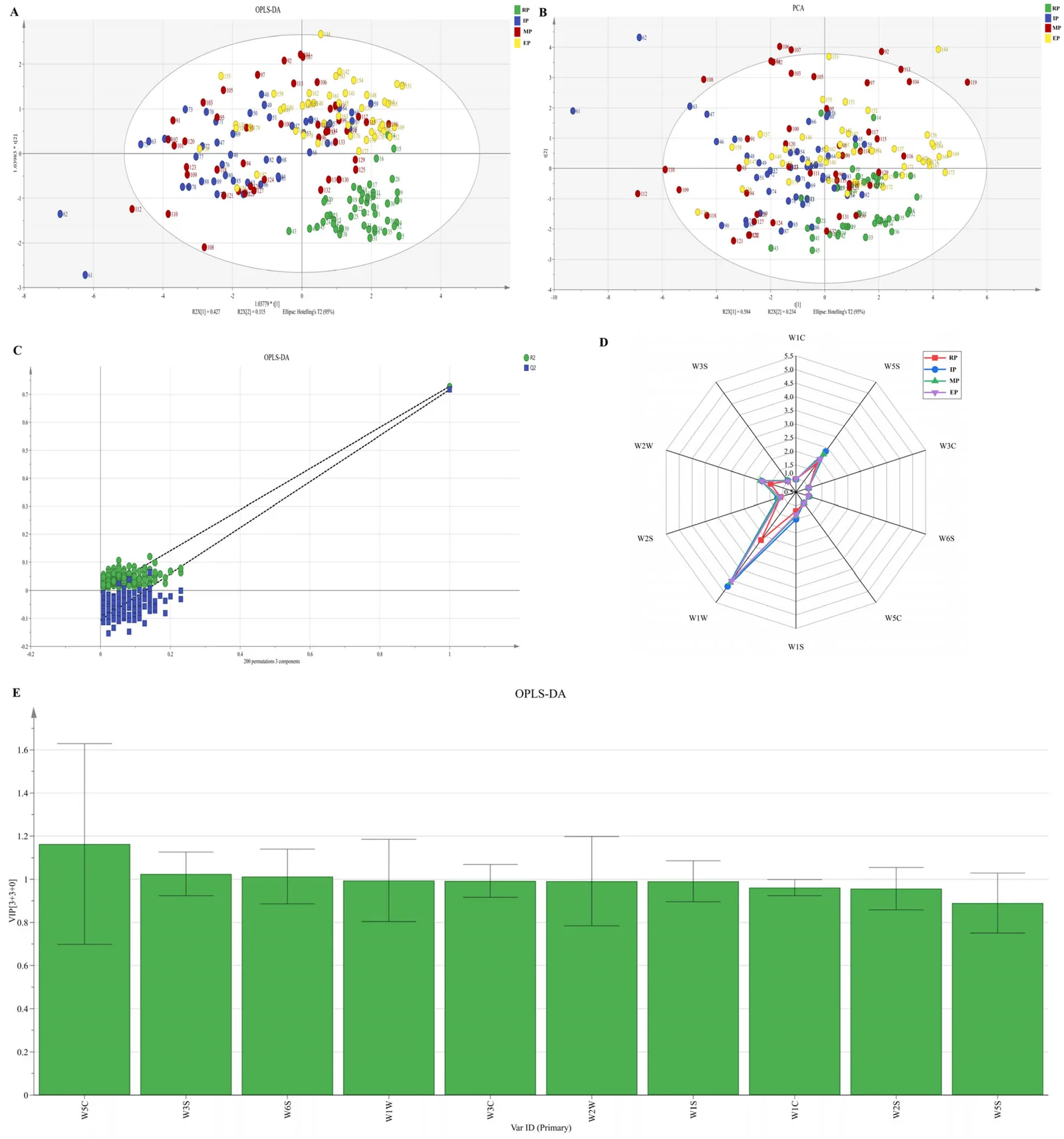
Experimental results of electronic nose. (**A**) Electronic nose PCA result chart. (**B**,**C**) Electronic nose OPLS-DA analysis result chart. (**D**) Electronic nose result radar chart. (**E**) VIP value diagram of electronic nose sensor.

The electronic nose detected differences in odor responses across various processing stages of fired CF. However, multivariate statistical analysis using PCA and OPLS-DA (Figure 2A,B) revealed significant overlap among sample points from the four processing degrees, with blurred category boundaries, failing to achieve effective differentiation. Within the sensor array, specific peaks were observed for W5S (sensitive to nitrogen oxides), W1W (sensitive to sulfides), and W2W (sensitive to alcohols/aldehydes/ketones) in the appropriately processed products (Figure 2D). This suggests the generation and transformation of volatile substances, such as nitrogen oxides and organic sulfides, at this processing stage, consistent with Maillard and pyrolysis reaction patterns. The variable importance in projection (VIP) plot presented in Figure 2E validated the core discriminatory role of these sensors (VIP value > 1). The system combines ten different metal oxide semiconductor sensors, as shown in Table S2.

### 3.3. Machine Learning-Based Classification Model

The internal cross-validation accuracy on the training set and prediction accuracy on the unseen independent test set of the six machine learning models are listed in Table 2, which intuitively reflects the fitting performance and generalization capacity of each model. Based on evaluations from the stratified hold-out split combined with internal 10-fold cross-validation (Table 3), all machine learning models demonstrated good discriminative capabilities in the four-class classification task concerning the processing degrees of fried CF. The superior classification performance achieved by the combined color and electronic nose feature set can be attributed to the complementary roles of the two information sources. Lab chromatic parameters directly reflect the surface browning degree induced by the Maillard reaction and caramelization during thermal treatment, functioning as intuitive visual indicators of stir-frying intensity, and exhibit particularly high discriminative power for raw products and severely over-processed samples with distinct appearance differences. Electronic nose sensor signals, by contrast, capture volatile metabolite profiles generated from lipid oxidation, trigonelline degradation, and sugar–amino acid cross-coupling reactions under sustained heating, revealing intrinsic chemical changes that cannot be identified from visual appearance alone, and enabling effective differentiation of samples with similar color but different internal processing states. The fusion of these two complementary data modalities covers both superficial visual attributes and inner chemical properties, compensating for the limitations of single-source information and contributing to the enhanced classification accuracy and robustness observed in all models. Ensemble models achieved promising classification performance for fried hemp seed processing identification. XGBoost obtained an overall accuracy of 0.9167 and a macro AUC of 0.9835, with balanced recall and an F1-score over 0.9167. While Logistic Regression delivered the maximum macro AUC of 0.9856, its linear structure limits its ability to capture nonlinear color–odor relationships. XGBoost and Logistic Regression achieve identical test accuracy (91.67%), while Logistic Regression obtains a marginally higher macro AUC. XGBoost is selected as the final classifier based on comprehensive multi-dimensional criteria rather than single predictive indicators. Native TreeSHAP interpretability is important, but not the only consideration. As an optimized gradient-boosting tree ensemble, XGBoost effectively models nonlinear cross-correlations within fused color–electronic nose features, maintains balanced classification performance across all four processing groups to reduce minority sample misclassification, and provides stronger anti-noise stability for on-site herbal quality monitoring. These practical strengths determine XGBoost as the optimal candidate for this task. Therefore, XGBoost was chosen for SHAP analysis to interpret feature importance, leveraging the efficient TreeSHAP algorithm for reliable and interpretable feature contribution quantification.

Considering classification performance, resistance to overfitting, computational efficiency, and good support for interpretability tools such as SHAP, XGBoost not only demonstrated optimal performance in this study but also possesses favorable scalability and interpretability, representing an ideal model choice for future work involving large-scale sample classification and the construction of intelligent processing quality evaluation systems.

### 3.4. Validation of the Classification Performance of Machine Learning Models

**Figure 3:**
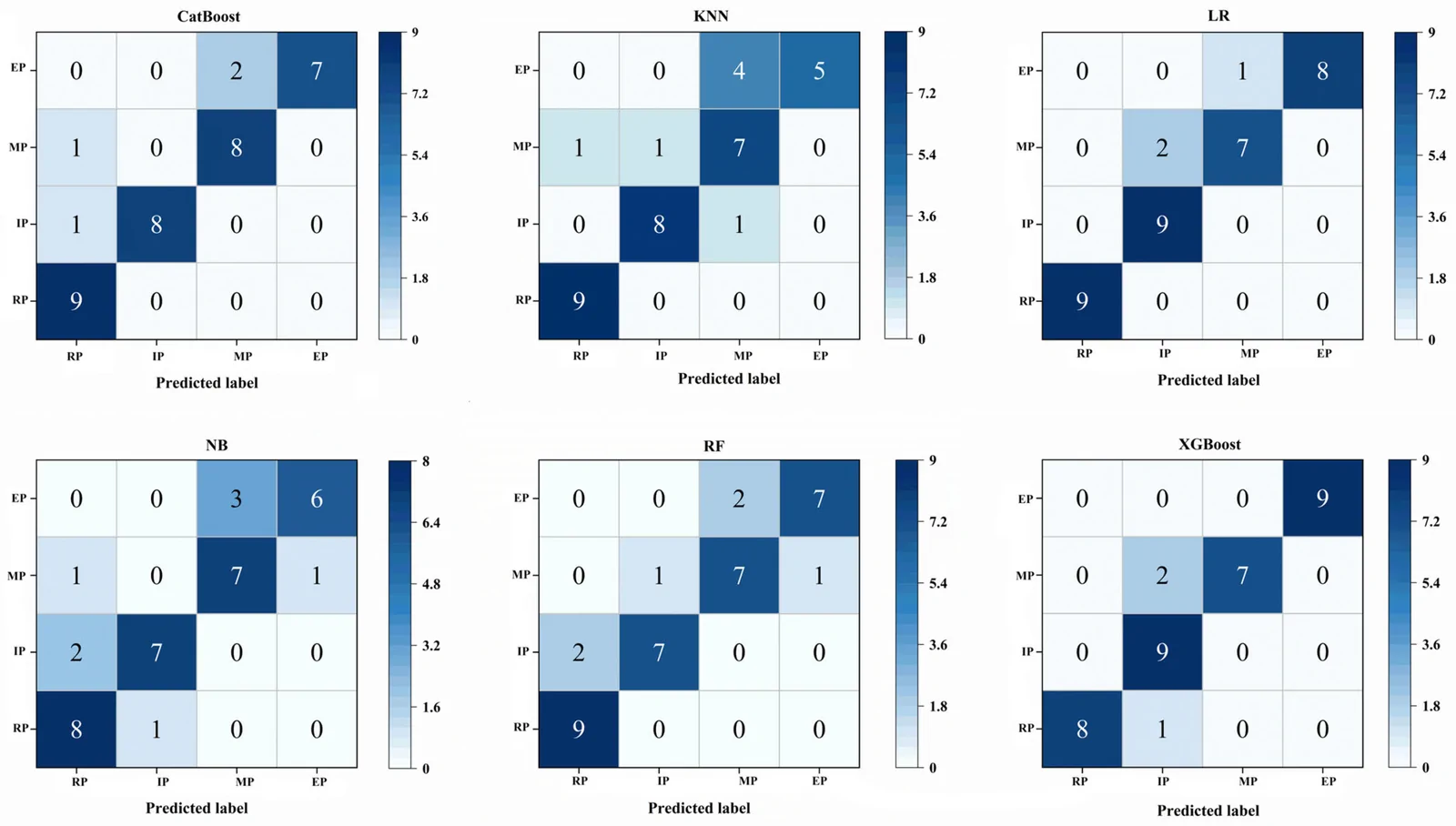
Performance comparison chart of each classification model.

To achieve accurate discrimination of different processing degrees of fried CF, this study constructed six machine learning classification models—CatBoost, RF, XGBoost, NB, KNN, and LR. The classification performance of each model was visualized using confusion matrices (Figure 3). In these matrices, the rows represent true sample labels, while columns represent the predicted labels from the model. The numerical value in each cell suggests classification counts for the corresponding category, with color depth positively correlated with count. The XGBoost model achieved 100% classification accuracy (9/9) for IP and EP. For RP, it correctly classified eight samples and misclassified one as the original product; for MP, it correctly classified seven samples and misclassified two as IP, demonstrating balanced overall classification performance. The CatBoost model achieved 100% accuracy (9/9) for RP; however, its classification error rates for IP and EP were slightly higher than those of XGBoost. Random Forest, KNN, and Logistic Regression produced noticeable cross-category misclassifications (e.g., two raw samples misclassified as moderately processed by Random Forest), reflecting weaker stability versus XGBoost. Alongside balanced prediction accuracy across all categories, XGBoost’s inherent algorithmic strengths for nonlinear fitting and SHAP compatibility supported its selection for subsequent interpretable feature analysis [30].

### 3.5. Interpretable Machine Learning: SHAP Feature Analysis

**Figure 4:**
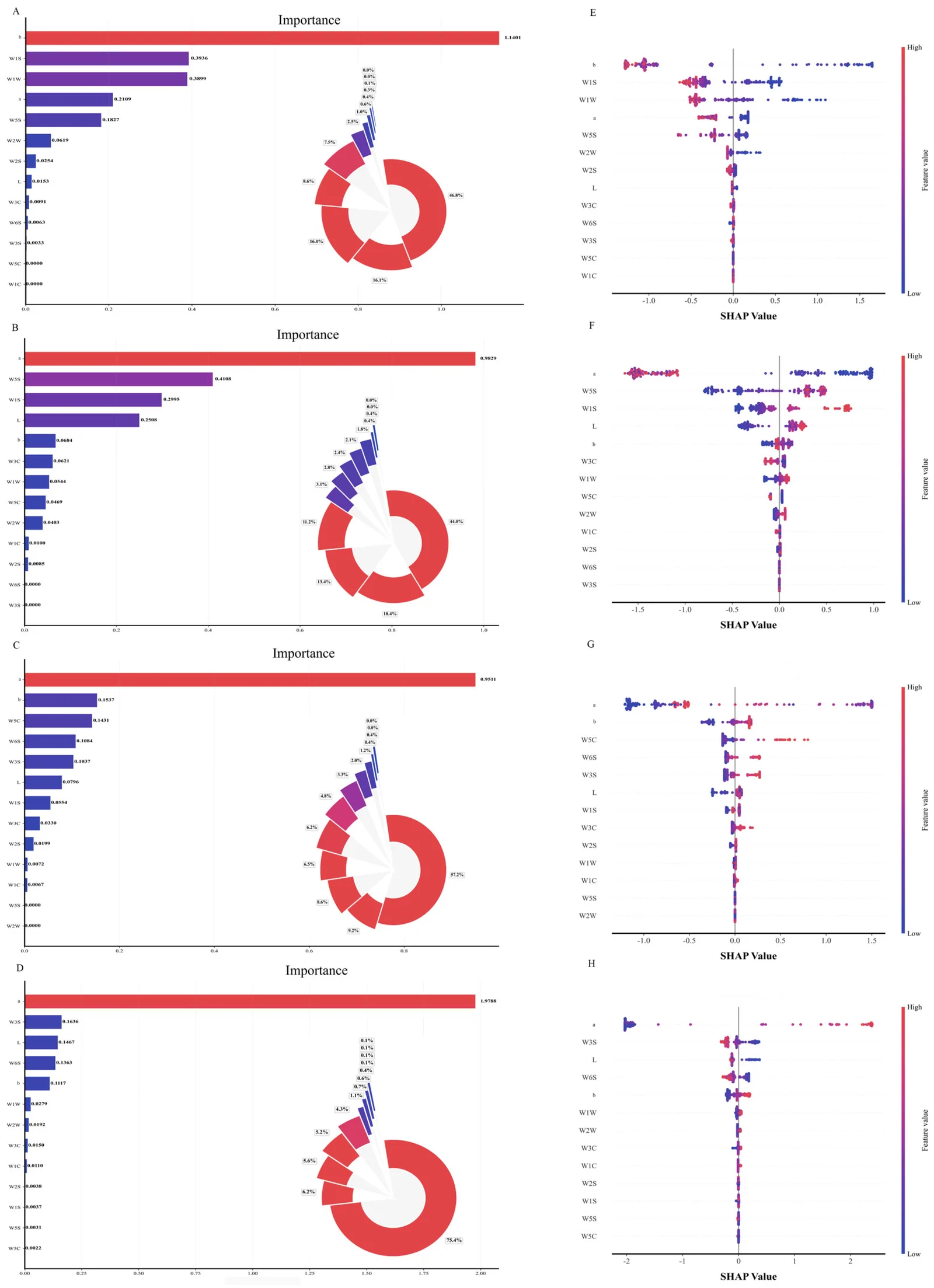
The contribution degree of sensory information at different processing levels using the SHAP method. (**A**,**E**) RP SHAP summary plot; (**B**,**F**) IP SHAP summary plot; (**C**,**G**): MP SHAP summary plot; and (**D**,**H**) EP SHAP summary plot.

To enhance decision transparency and the credibility of the classification model results, this study adopted the overall best-performing XGBoost model and introduced the SHAP method. Multi-dimensional visualizations revealed the importance of features and their contribution mechanisms to the discrimination of processing degrees (Figure 4).

#### 3.5.1. SHAP-Based Global Feature Importance and Underlying Thermal Reaction Mechanisms

The SHAP global feature importance ranking indicated that Lab color space parameters and the responses of key electronic nose sensors (a, b, W1S) collectively constitute the core discriminative features of the classification model (bar charts on the left side of Figure 4A–D). In the bar chart, higher feature importance scores indicate stronger influence on the model’s classification decision. The accompanying donut charts further quantify the proportional contribution of each feature’s importance [31,32]. For instance, in the RP group, the b value (yellow–blue chromaticity) accounted for 46.8% (Figure 4A), whereas in the IP, the W5S sensor contributed 18.4% (Figure 4B), offering an intuitive visualization of the differences in feature weights across processing degrees. The top-ranked discriminative indicators extracted via SHAP analysis include Lab color parameters (L, a, b) and critical electronic nose sensor signals (W1S, W1W, W5S), each carrying clear physicochemical significance tied to stir-frying thermal reactions. These sensory changes are consistent with general thermal reaction patterns widely reported in seed roasting studies [33]. The L value represents sample brightness, which gradually decreases with extended heating as the Maillard reaction and caramelization continuously generate brown melanoidin polymers, darkening the surface of CF. Parameters a and b capture red–yellow chromatic transitions originating from endogenous chlorophyll degradation and the generation of colored thermal by-products [34]. Among electronic nose sensors, W5S, W1W, and W1S appear to be the three most influential odor discriminators according to SHAP global importance ranking, and their relatively high feature weights may stem from characteristic thermal chemical reactions occurring during stir-frying, consistent with documented thermal transformation trends of seed-derived components. W5S, sensitive to nitrogen oxides, may capture nitrogen-containing volatiles formed through Maillard sugar-amine condensation and trigonelline thermal degradation; its signal intensity tends to reach a maximum at the moderate frying stage, where nitrogenous intermediates likely accumulate before further breakdown under sustained high heat. W1W, selective for sulfur-containing organics, possibly detects volatile sulfides yielded by pyrolysis of endogenous sulfur amino acids in CF, and such flavor-related compounds may gradually build up as heating time extends [35]. W1S, responsive to methyl, aldehyde, and ketone substances, might reflect carbonyl by-products from lipid oxidation and early Maillard intermediates, which could correspond to the gradual release of total fatty oil during stir-frying. W2W, targeting aromatic sulfides, may also offer auxiliary discriminative capacity by capturing secondary pyrolysis substances likely generated under over-heated conditions. The distinct feature contribution weights across raw, under-processed, moderately stir-fried, and over-processed samples could mirror the dynamic formation, accumulation, and degradation of these heat-induced compounds under different thermal intensities, which may help establish a tentative sensory correspondence to the physicochemical shifts taking place throughout stir-frying.

#### 3.5.2. Category Specificity of Feature Contribution

Figure 4 presents a beeswarm plot, illustrating the relationship between each feature’s value and its corresponding SHAP value. A larger absolute SHAP value for a feature suggests greater influence of that feature on the CatBoost-based classification model [36,37,38]. This visualization reflects the magnitude of each feature’s impact. The color gradient ranging from blue (low) to red (high) represents the raw value of individual features. The absolute SHAP value quantifies the strength of each feature’s effect on model output, whereby larger values correspond to greater influence. The feature with the highest importance displays the broadest SHAP value distribution. For this primary feature, high feature values were predominantly associated with negative SHAP values, meaning elevated levels of this feature generally imposed a negative effect on the model output of the target class. In contrast, features located near the bottom of the beeswarm plot exerted relatively weak effects on classification.

Further explanation was provided by the SHAP plots for groups RP, IP, MP, and EP, which illustrate the specific influence of each indicator on each group. For RP (Figure 4A,E), b, W1S, and W1W are the three most important features: the samples corresponding to high values of b are mostly distributed in the negative SHAP interval, indicating that an increase in b values will reduce the probability of samples belonging to the target category. The W1S and W1W scatter points span the positive and negative SHAP intervals, and exhibit bidirectional contribution characteristics.

For IP (Figure 4B,F), a, W5S, and W1S are the three most important features. Low-level a samples often fall in the negative SHAP interval, which tends to reduce the probability of samples belonging to the target category. High-value sample sets of W5S and W1S are distributed in the positive SHAP interval, which is beneficial for improving the probability of target category attribution.

For MP (Figure 4C,G), a, b, and W5S are three important features. A low level of a tends to reduce the probability of samples belonging to the target category, while a high value of a can significantly increase this probability. b and W5C exhibit similar regulatory patterns.

For EP (Figure 4D,H), with higher values of a, L, and W3S, a low level of a tends to reduce the probability of samples belonging to the target category, while a high value of a can significantly increase this probability. W3S and L exhibit bidirectional nonlinear regulatory effects [38].

### 3.6. Key Driving Factors and Critical Values

**Figure 5:**
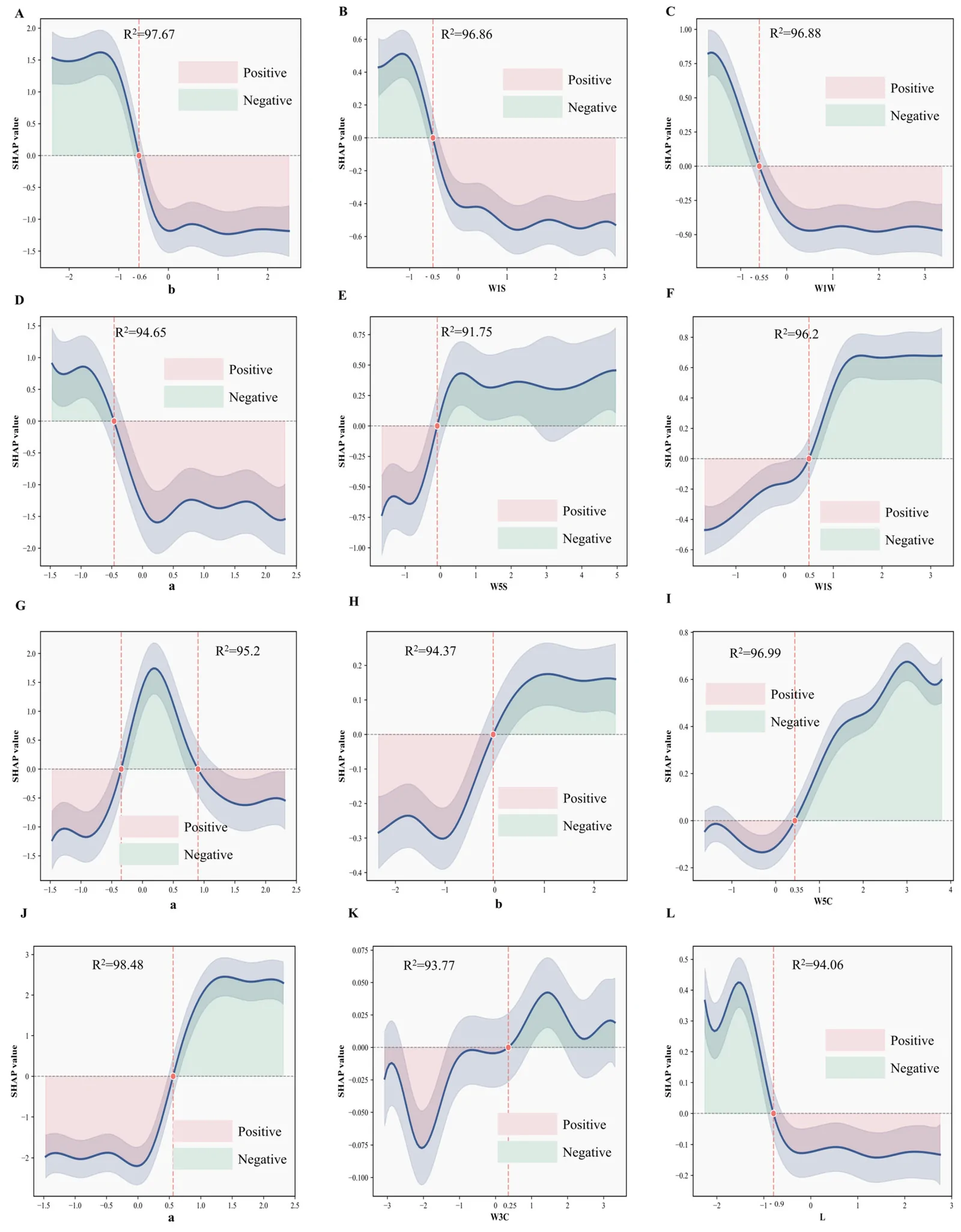
Identification of critical points for key factors in different processing levels. (**A**–**C**) The key parameter range of RP. (**D**–**F**) The key parameter range of IP. (**G**–**I**) The key parameter range of MP. (**J**–**L**) The key parameter range of EP.

Based on core drivers identified through SHAP feature analysis, this study examined response patterns of these features and combined them with dependence plots of the top three contributing features to uncover correlation patterns between each feature and processing degree. This analysis further clarified the critical intervals for key features, providing a digital reference for quality control in processing technology (Figure 5).

#### 3.6.1. Identification of Critical Thresholds for Individual Factors

For RP (Figure 5A–C), the model had a high confidence in determining RP when the value of b was between −0.6 and 2.3; the confidence sharply declines when it falls between −2.2 and −0.6. For the W1S sensor value, the critical range was from −0.5 to 3.1, where the model exhibited the highest prediction confidence for classifying a sample as RP. However, confidence decreases sharply when the value falls between −2.1 and −0.5. For the W1W sensor value, the critical range was from −0.55 to 3.15, where the model exhibited the highest prediction confidence for classifying a sample as RP. The confidence decreases sharply when the value falls between −2.1 and −0.55.

Regarding the b value, the model had the highest confidence in predicting RP when it ranged from −0.6 to 2.3, although confidence sharply declined when the value was between −0.22 and −0.6. This phenomenon is likely closely related to intrinsic components and the physical state of SP: the seed coat of raw CF contains stable chlorophyll and inherent pigments (chlorophylls, carotenoids), while the generation of nitrogen-containing volatile compounds is minimal, corresponding with the characteristic negative color value interval and low odor response [39,40].

For IP (Figure 5D–F), the model had the highest confidence in predicting IP when the a value ranged from −0.5 to 2.4, while confidence declined sharply for values between −1.5 and −0.5. For W5S sensor values, the critical range was from −1.8 to −0.15; within this range, the model exhibited the highest prediction confidence for IP, with confidence sharply dropping when the value fell between −0.15 and 5. A W1S sensor value from −0.15 to 0.5 yielded the highest confidence in predicting IP, with confidence dropping sharply between 0.5 and 3. This decline in prediction confidence is likely due to incomplete thermal reactions involving partial degradation of chlorophyll and initial generation of nitrogen- and sulfur-containing volatile compounds, resulting in features characteristic of the transitional state between raw and appropriately processed products [41].

For MP (Figure 5G–I), the confidence level of MP is determined to be the highest within the range of −1.5 to −0.4 and 0.85 to 2.4 for model a value; when the value is between −0.4 and 0.85, confidence drops sharply. For b, the model exhibited the highest confidence in determining MP when the value was within −2.4 to 0, with confidence dropping sharply for b values between 0 and 2.5. For W5C sensor values, the model had the highest confidence in determining MP within the range of −1.8 to 0.35, with confidence dropping sharply between 0.35 and 3.8. This is likely attributed to the moderate degradation of chlorophyll and the Maillard reaction reaching an equilibrium state, leading to the peak generation of yellow intermediates and volatile flavor compounds (aldoketones) [42].

For EP (Figure 5J–L), the model achieved the highest confidence in determining EP when a fell within −1.5 to 0.55; a confidence drop occurred between 0.55 and 2.5. For W3C, peak confidence for classifying samples as EP occurs within −3 to 0.25, decreasing between 0.25 and 3.5. For L values, confidence remains highest between −0.9 and 2.8, with a sharp decline within −2.3 to −0.9, potentially attributed to excessive browning reactions (Maillard reaction/caramelization) during over-processing, culminating in brownish substance accumulation and the degradation of trigonelline, thereby aligning positive intervals for a with negative intervals for L.

#### 3.6.2. Multi-Factor Combined Interval Characteristics

Combined feature analysis revealed that specific feature intervals exhibited matching correlations. Specifically, the combined interval where b is between −0.6 to 2.3 and W1S is within −0.5 to 3.1 corresponds to color characteristics of RP. The combined interval containing the range of a sensor values from −0.5 to 2.4 and the range of W5S sensor values from −1.8 to −0.15 corresponds to odor characteristics of IP. For MP, when a falls between −0.45 to 0.8 and 0.85–2.4, the b value is within −2.4 to 0; color characteristics were evident. When a is within −1.5 to 0.55, the critical values of b are between −3 to 0.25 and for L between −0.9 to 2.8; the corresponding color characteristics of the product were quantified. This feature captures the shape- and color-related features pertinent to traditional processing experiences and clarifies the matching range between color and odor characteristics. This finding provides reference data for refining the parameters of the processing technique [43].

### 3.7. Determination of Principal Constituents in CF with Different Processing Degrees

#### Determination of Intrinsic Component Content

**Figure 6:**
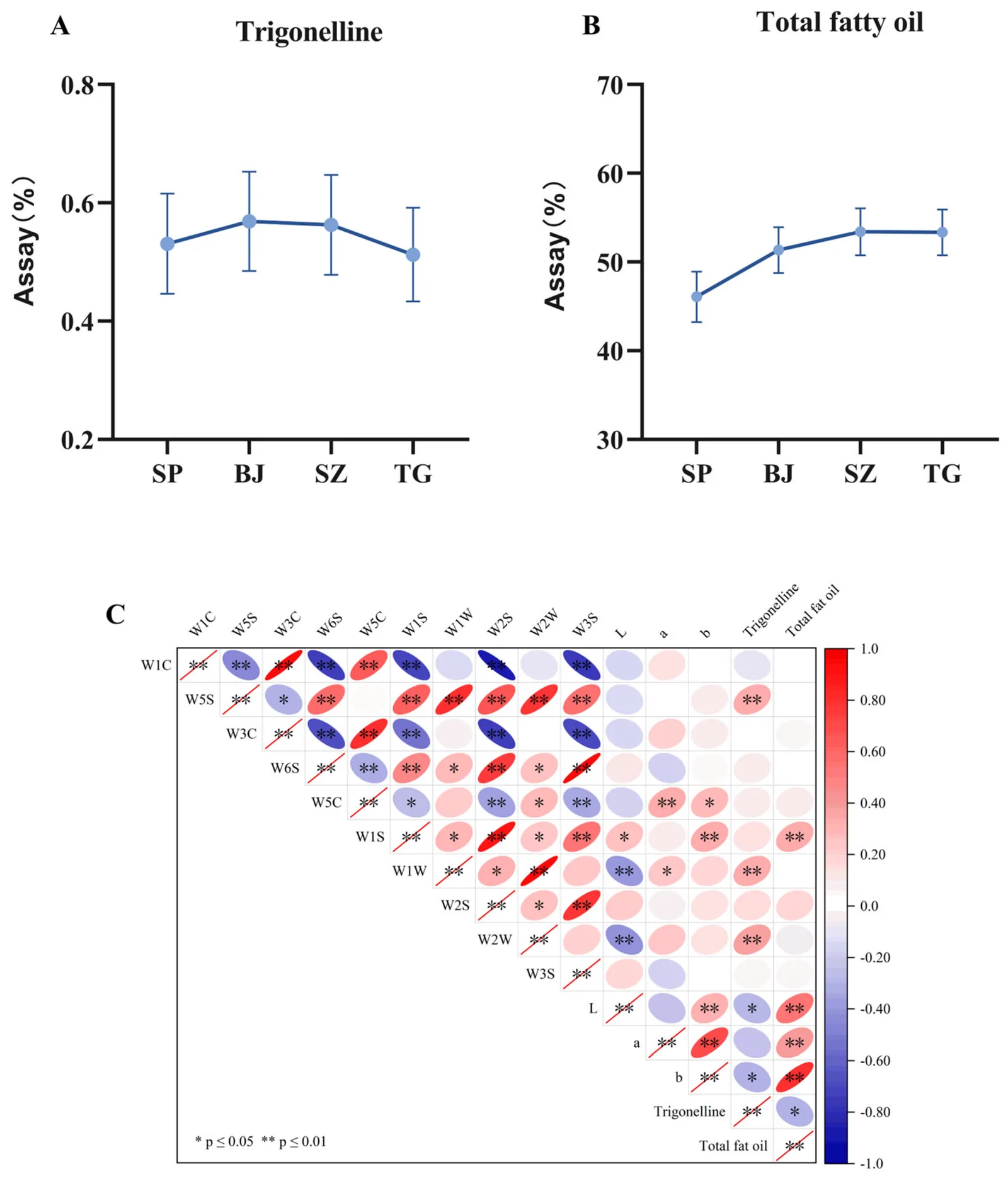
Correlation diagrams of different senses and internal components. (**A**) Trigonelline variation graph. (**B**) Total fatty oil variation graph. (**C**) Correlation graph.

After processing, there were variations in trigonelline content in CF specimens, as determined by high-performance liquid chromatography (HPLC) (Figure 6A). Trigonelline content in IP and MP CF was similar, both showing an increase compared to levels in RP. In contrast, EP CF exhibited decreased trigonelline content relative to RP. This reduction may be attributed to prolonged stir-frying initiating the thermal decomposition of trigonelline [44].

The total fatty oil content of fried CF pieces across four processing degrees is presented in Figure 6B. The table shows a substantial change rate in total fatty oil content relative to RP for each processing degree. Total fatty oil content in CF gradually increased with longer stir-frying duration and tended to stabilize following appropriate processing stages.

### 3.8. Correlation Analysis Between Intelligent Sensory Data and Intrinsic Components

Spearman correlation analysis was conducted to explore the relationships between the contents of total fatty oil and trigonelline, as well as color and odor indicators of stir-fried CF (Figure 6C). To a certain extent, total fatty oil tended to increase with rising L and b values, and statistically significant positive correlations were detected for both of the two parameters. By contrast, trigonelline content presented a weak negative tendency against L and b, which suggested that samples with lower brightness and weaker yellow tone might contain relatively higher trigonelline levels. Spearman correlation analysis demonstrated an interrelationship between the color of fried CF and its total fatty oil and trigonelline contents, suggesting that appearance color could, to a certain extent, serve as an indicator for estimating levels of total fatty oil and trigonelline. Among electronic nose sensors, W5S, W1W, and W2W showed the highest odor responses to fried CF across differing processing degrees. Specifically, the responses from W5S, W1W, and W2W exhibited relatively high correlations with trigonelline levels, while the W1S sensor response showed a relatively high correlation with total fatty oil content. Notably, statistical significance confirms the reliability of the observed association trends, but the practical quantitative inference value of the correlation coefficients requires objective evaluation. The correlation coefficients identified in this study are mostly at weak-to-moderate levels. This pattern is consistent with the complex multi-reaction characteristics of the stir-frying process: Lab color parameters and electronic nose signals are comprehensive outputs of multiple concurrent thermal reactions, including non-enzymatic browning, fatty oil oxidation, and endogenous component degradation. A single sensory indicator is therefore unlikely to exhibit a strong linear correlation with one individual chemical constituent, and precise quantitative prediction of component content solely from sensory data is inappropriate. Nevertheless, these statistically significant correlations still deliver practical reference value for rapid preliminary quality screening. Non-destructive sensory signals can serve as surrogate indicators to reflect general quality variation trends across batches, reducing the demand for full-component chemical testing in routine preliminary screening scenarios.

Regarding the model-building validation scheme applied in the present study, the dataset splitting strategy deserves further clarification. The three parallel samples within each batch serve as independently prepared stir-frying process replicates encompassing normal process variations during raw material processing and heating procedures; since they are not identical technical replicates of the same sample, there is no underlying basis for information leakage associated with splitting the same sample into both training and test datasets. Inclusion of such normal intra-batch process variations in the modeling dataset aligns with the conventional practice of production quality control, as enabling the model to adapt to routine process fluctuations in actual production during the training phase improves the robustness and practical applicability of the classification system in real-world scenarios and represents a rational design oriented to practical production implementation. Accordingly, no information leakage risk is present in the adopted validation strategy, and this approach can effectively ensure the stability of model training and the fairness of head-to-head comparison across the six algorithms. Batch-level cross-validation with expanded raw material batches will be adopted in future work to more rigorously verify model generalizability for completely new production batches. Additionally, the generalizability of the model under real industrial production conditions requires further validation. Furthermore, the present investigation exhibits two distinct merits relative to existing relevant literature. Prior studies largely confine their analytical scope to the establishment of classification models relying merely on sensory features, with limited exploration of their linkage to intrinsic quality constituents and model interpretability. This work constructs a “color–odor–quality” correlation system consistent with traditional Chinese medicine quality evaluation theories by associating multi-source sensory information with two core quality indicators of stir-fried CF. Grade-specific SHAP analysis is performed to quantify the individual contribution of each feature and extract corresponding critical ranges, rendering model predictions interpretable and delivering theoretical and data support for the optimization of stir-frying technological parameters as well as real-time online monitoring in industrial processing.

Statistically significant correlations between Lab color features, electronic nose signals, and intrinsic chemical markers offer preliminary guidance for stir-fried CF quality monitoring. Unlike laborious offline gravimetry and HPLC, utilizing machine vision and an electronic nose enables fast, non-destructive, sensory fingerprint acquisition to track changes in total fatty oil and trigonelline, cutting full routine chemical testing for batch pre-screening. The SHAP-derived critical feature intervals are generated under controlled laboratory settings and only supply lab-scale conceptual data, rather than ready-to-use industrial online monitoring standards. Raw material origin differences and unstable furnace temperatures in real production demand further calibration of these laboratory thresholds, and large industrial sample validation is required to optimize feature intervals for reliable on-site application.

Comparisons with published studies on machine vision, electronic nose use, and machine learning for thermally processed food and herbal decoction pieces reveal consistent experimental patterns. Separate color or odor sensing data cannot achieve satisfactory classification of different frying degrees, while multi-source fusion of the two types of sensory data can effectively improve model discrimination, which matches our findings. Most existing relevant studies only focus on constructing classification models based on sensory features, with little discussion on the correlation between sensory signals and intrinsic chemical compositions of raw materials, as well as model interpretability. Different from these works, this study simultaneously detects two core quality components of *Cannabis fructus*, total fatty oil and trigonelline, and introduces grade-targeted SHAP analysis to quantify feature contributions and extract feature threshold ranges, which provides supplementary analytical perspectives for similar research frameworks. The classification accuracy and AUC of the XGBoost model in this paper are comparable to those of small-sample laboratory studies on fired seed products. Similar to most laboratory-scale research, the feature intervals summarized herein still need further verification with industrial batches to enhance the generalization performance of the model in actual production.

In addition, only total fatty oil and trigonelline were quantified in this experiment, without detection of volatile substances, cannabinoids, phenolics, and other characteristic components. This incomplete component profiling limits the comprehensive biochemical interpretation of the correlations between sensory signals and classification results; multi-index metabolomics analysis will be supplemented in subsequent studies.

## 4. Conclusions

This study evaluated the performance of six machine learning algorithms and selected the one with the highest comprehensive score. By integrating explainable artificial intelligence techniques, such as SHAP analysis, an intelligent classification model for the processing degrees of fired CF was constructed based on the fusion of multi-source information, including color (Lab) and electronic nose data. The study revealed the changing patterns and intrinsic relationships between “color–odor–quality” across different processing stages. The findings indicate that during the stir-frying process, sample color transitioned from a light to a yellowish tint, closely associated with the Maillard reaction. The response values of electronic nose sensors W5S, W1W, and W2W peaked during the appropriately processed stage, suggesting the synergistic formation of nitrogen, sulfur, and carbonyl-containing volatile substances. Correlation analyses further indicated that total fatty oil content showed a significant positive correlation with L and b values; in contrast, trigonelline content decreased with over-processing and was significantly associated with specific odor signals. XGBoost emerged as the model demonstrating the best classification performance in the four-class task, achieving accuracy, recall, and AUC values of 0.9167, 0.9167, and 0.9835, respectively. SHAP analysis quantified the contribution of different features to classification results, identifying the L value, b coordinate, and signals from sensors W5S and W1W as key features influencing classification decisions. This offers an interpretable physical basis for model predictions. The “multi-source information fusion–machine learning–SHAP interpretation” framework established in this study not only facilitates rapid and objective identification of the processing degree of fried CF, but also provides reliable methodological support for intelligent monitoring and digital quality evaluation of similar processing procedures for other Chinese herbal products.

## Figures and Tables

**Table 1:** The hyperparameters and corresponding optimal values of the six machine learning models.

Model Name	Parameter	Optimal Value
CatBoost	depth	4
	L2_leaf_reg	2
	learning_rate	0.036784184
KNN	n_neighbors	6
	P	4
XGBoost	n_estimators	612
	max_depth	2
	learning_rate	0.02891889
	subsample	0.552373384
Logistic Regression	C	0.132929
	penalty	None
	solver	saga
Naive Bayes	var_smoothing	1 × 10^−10^
Random Forest	max_depth	8
	max_features	log2
	min_samples_leaf	2
	min_samples_split	6

**Table 2:** Training and test sets of the six classification models.

Model Name	Optimal Accuracy of 10% off CV Within the Training Set	Independent Test Set Accuracy
CatBoost	0.964286	0.888889
KNN	0.902857	0.805556
XGBoost	0.93619	0.916667
Logistic Regression	0.944762	0.916667
Naive Bayes	0.854762	0.777778
Random Forest	0.923333	0.833333

**Table 3:** Evaluation results of stratified hold-out split combined with internal 10-fold cross-validation.

	CatBoost	NB	KNN	Logistic	XGBoost	RF
Accuracy	0.8889	0.7778	0.8056	0.9167	0.9167	0.8333
Precision	0.9045	0.7899	0.8431	0.9233	0.9375	0.8365
Recall (Sensitivity)	0.8889	0.7778	0.8056	0.9167	0.9167	0.8333
F1-score	0.8896	0.7776	0.8043	0.9162	0.9183	0.8312
Specificity	0.963	0.9259	0.9352	0.9722	0.9722	0.9444
AUC	0.9835	0.9414	0.9486	0.9856	0.9835	0.9784

Note: For the four-class classification, the F1-score denotes macro-F1, calculated as the arithmetic mean of F1-scores for each class.

## Data Availability

The original contributions presented in this study are included in the article/Supplementary Material. Further inquiries can be directed to the corresponding authors.

## Supplementary Materials

The following supporting information can be downloaded at: https://www.mdpi.com/article/10.3390/foods15162817/s1; Table S1: Information on 15 batches of stir-fried CF slices. Table S2: Electronic nose system sensor type and performance description.

## Abbreviations

The following abbreviations are used in this manuscript:

CF, *Cannabis fructus*; RP, Raw product; IP, Insufficient frying degree product; MP, Moderate frying degree product; EP, Excessive degree of frying product; SHAP, Shapley Additive Explanations; HPLC, High-performance liquid chromatography; Lab, CieLab; L, Luminosity; a, Green to Red; b, Blue to Yellow; CatBoost, Categorical Boosting; XGBoost, Extreme Gradient Boosting; NB, Naive Bayes Classifier; RF, Random Forest; KNN, K-Nearest Neighbor; LR, Logistic Regression.
