# Supplementary material for: An Intelligent Identification Method for the Processing Degree of Stir-Fried Cannabis fructus Based on Multi-Source Information Fusion and Machine Learning
Source: Foods. 2026 Aug 12;15(16):2817. doi: 10.3390/foods15162817 (PMC13512757; doi:10.3390/foods15162817)
Supplement: Supplementary file 1 [file foods-15-02817-s001.zip › foods-4493731-supplementary.pdf]

Table S1 Information of 15 Batches of Stir-fried CF Slices

Number	Lot number	Source
1	20230608018	Shanxi
2	20230608019	Shanxi
3	20230608020	Shanxi
4	20230608021	Shanxi
5	20230608022	Shanxi
6	20230608023	Henan
7	20230608024	Henan
8	20230608025	Henan
9	20230608026	Henan
10	20230608027	Henan
11	20230608028	Heilongjiang
12	20230608029	Heilongjiang
13	20230608030	Heilongjiang
14	20230608031	Heilongjiang
15	20230608032	Heilongjiang

Table S2 Electronic nose System Sensor Type and Performance Description

Array number	Sensor name	Performance description
1	W1C	Aromatic benzene
2	W5S	Nitrogen oxides
3	W3C	Aromatic ammonia
4	W6S	Hydrogen
5	W5C	Alkane aromatic compounds
6	W1S	Chain alkanes
7	W1W	Sulfides and terpenes
8	W2S	Alcohols
9	W2W	Organic sulfides and aromatic components
10	W3S	Long-chain alkanes
